# Optimization of thermoelectric performance of SrSi_2_-based alloys via the modification in band structure and phonon-point-defect scattering

**DOI:** 10.3389/fchem.2014.00106

**Published:** 2014-11-25

**Authors:** Yung-Kang Kuo, Balakrishnan Ramachandran, Chin-Shan Lue

**Affiliations:** ^1^Department of Physics, National Dong Hwa UniversityHualien, Taiwan; ^2^Department of Physics, National Cheng Kung UniversityTainan, Taiwan

**Keywords:** metal silicides, eco-friendly thermoelectric material, optimization of *ZT* value, charge carriers doping, band engineering, point-defect scattering

## Abstract

Thermoelectric properties of alkaline-earth-metal disilicides are strongly dependent on their electronic band structure in the vicinity of the Fermi level. In particular, the strontium disilicide, SrSi_2_ with a narrow band gap of about few tens of meV is composed of non-toxic, naturally abundant elements, and its thermoelectric properties are very sensitive to the substitution/alloying with third elements. In this article, we summarize the thermoelectric performance of substituted and Sr-deficient/Sr-rich SrSi_2_ alloys to realize the high thermoelectric figure-of-merit (*ZT*) for practical applications in the electronic and thermoelectric aspects, and also to explore the alternative routes to further improve its *ZT* value.

## Introduction

Over the past decades, there has been a vast interest in thermoelectric materials to attain sustainable electrical energy through the handling of waste heat by thermoelectric generators. In general, thermoelectric generators are heat engines that utilize the thermoelectric or Seebeck effects (Newnham, 2005). In the early development of thermoelectric applications, the performance of devices made of metals was disappointing due to its small Seebeck coefficient and large thermal conductivity. However, the situation changed with the discovery of much higher Seebeck coefficients in semiconductors. Generally, home heating, automotive exhaust and industrial processes all generate an enormous amount of unused waste heat that can be converted to electrical energy using thermoelectrics (Snyder and Toberer, 2008). As thermoelectric generators are solid-state devices with no moving parts, they are silent, reliable and scalable, which makes them an ideal tool for small scale power generation (Rowe, 1995). Efforts are already underway to replace the alternator in cars with a thermoelectric generator in the exhaust stream, to improve the fuel efficiency (Matsubara, 2002).

By utilizing Seebeck effects, thermoelectric materials can generate electricity from the waste heat of automobile exhaust and various industrial processes (Wan et al., 2010). However, currently available thermoelectric materials are not effective for most practical applications. The efficiency of thermoelectric materials can be estimated by the figure-of-merit, *ZT*(= *S*^2^*T*/ρκ), where *S*, ρ, and κ represent the Seebeck coefficient, the electrical resistivity and the thermal conductivity, respectively. In general, thermal conductivity (κ) is simply a sum of the lattice component (κ_*L*_) and the electronic component (κ_*e*_), according to the Wiedemann–Franz law, κ_*e*_(*T*)ρ(*T*) = *L*_0_*T*, where *L*_0_ (= 2.45 × 10^−8^ WΩK^−2^) is the Lorenz number. The record dimensionless figure-of-merit, *ZT_max_* = 2.4 has been attained in the artificial superlattice Bi_2_Te_3_-Sb_2_Te_3_ materials at 300 K (Venkatasubramanian et al., 2001), however it can be hardly used in thermoelectric generators because of their thermodynamic instability at high temperatures. In spite of many reports on very efficient thermoelectrics (Ettenberg et al., 1996; Polvani et al., 2001), the maximum dimensionless thermoelectric figure-of-merit of *n*-type materials used in commercial generator applications does not exceed unity above 600 K. In order to enhance the *ZT* value, the concept of phonon-glass electron-crystal was proposed, which states that the phonons transport (lattice thermal conductivity) should be suppressed as in glasses, and the electrical conductivity should be maintained as in crystals (Wan et al., 2010). Most recent development in thermoelectric materials has been made by nanostructuring to reduce the lattice thermal conductivity. The low dimension approaches gives potential new directions in thermoelectrics, however, there are some disadvantages of these materials. For examples, the superlattices or quantum dots structures are expensive and hard to reproduce, and they are too small to be used in routine industry applications. Besides, the figure-of merit of these complex systems is also found to be less than unity in most of recent reports (Shelimova et al., 2004; Zemskov et al., 2004; Kanatzidis, 2005). Even though many of these complex compounds have a low lattice thermal conductivity, their power factors (*PF* = *S*^2^/ρ) have not yet been optimized with proper doping. On the other hand, bulk intermetallic compounds have the advantage over the low-dimensional or bulk nano-composite materials as they are extremely stable, cost effective and easy to fabricate. Particularly, the optimization of band structure of a bulk material could result in a better figure-of-merit with the same electrical mobility, which was efficiently achieved in the bulk thermoelectric compound Mg_2_Si_1 − *x*_Sn_*x*_ (Zaitsev et al., 2006).

### Lattice thermal conductivity

Thermal transport in a solid is the dissipation of vibrational energy between adjacent atoms through chemical bonds (Wan et al., 2010). However, the heat transport (κ_*L*_ ∝ *C_v_lv*, where C_*v*_, *l*, and *v* are the heat capacity, the phonon mean free path and the phonon velocity, respectively) is complicated due to the broad spectrum of phonon frequencies in a solid. High-energy optical phonons do not have a sufficiently high group velocity to transport a substantial quantity of heat, and thus the thermal transport is often dictated by the longer-wavelength acoustic phonons (Wood, 1988). Usually, both *C_v_* and *v* are related to the intrinsic properties for a given material and are almost insensitive to the structural changes induced in the system. However, the phonon mean free path (*l*) can be affected by various scattering processes (Wan et al., 2010). Using the Debye model (Lue et al., 2004), the phonon thermal transport of thermoelectric materials can be described by the equation

(1) $$\kappa_{L}=\frac{k_{B}}{2\pi^{2}v}{\left(\frac{k_{B}T}{\hbar }\right)}^{3}\int_{0}^{\theta_{D}/T}\frac{\xi^{4}e^{\xi }}{\tau_{p}^{-1}{\left(e^{\xi }-1\right)}^{2}}d\xi ,$$

where ξ = *ħ*ω/*k_B_T* is dimensionless, *ħ*, ω, θ_*D*_ and τ^−1^_*p*_ are the reduced Planck's constant, the phonon frequency, the Debye temperature, and the phonon scattering relaxation rate, respectively. Here, τ^−1^_*p*_ is the combination of three scattering mechanisms as given below

(2) $$\tau_{p}^{-1}=\frac{v}{L}+A\omega^{4}+B\omega^{2}T\exp\left(\frac{-\theta_{D}}{3T}\right),$$

where *v* is the average phonon velocity, *L* is the grain size (comparable to the smallest sample dimension), and the coefficients *A* and *B* are the free-fitting parameters. The terms in Equation (2) are the scattering rates for the grain-boundary, point-defect, and phonon-phonon scatterings, respectively.

#### Grain boundary, point-defect, and Umklapp scatterings

Generally, the grain boundary scattering is a dominant mechanism for lattice thermal conductivity at low temperatures. While the scattering of phonons at the crystal boundaries has been known for a long time, it was previously thought to be a low-*T* phenomenon (Slack, 1979). Now, it is recognized that the boundary scatterings may also occur at higher temperatures and larger grain sizes. Indeed, the boundary scattering sometimes has a greater effect on lattice thermal conductivity than on carrier mobility, although the mean free path is larger for electrons/holes than that of phonons. The boundary scattering can be enhanced by the substantial contribution of low-frequency phonons (although their number is small) due to their long mean free path (Goldsmid and Penn, 1968).

Unit cells can be distorted with the introduction of point defects by substitution of foreign atoms into the lattice of materials. Point defects can scatter phonons, since they produce local variations in the sound velocity through change in density or elastic constants of the material. The relaxation time for such scattering should vary as 1/ω^4^. Thus, one can expect the low-frequency phonons will be influenced by point-defect scatterings (Tritt, 2004). Hence, the point-defect scattering could strongly affect the shape and position of the phonon peak, which usually found to appear at low temperatures.

The phonon-phonon scattering processes are resistive, and also known as Umklapp processes or U-processes. The total crystal momentum is not conserved for U-processes because these processes tend to restore the non-equilibrium phonon distribution to an equilibrium one, and give rise to thermal resistance (Tritt, 2004). However, other non-resistive and total momentum conserving processes in crystal (called as normal processes or N-processes) do not contribute to the thermal resistance, but they may still have strong influence on the lattice thermal conductivity of solids. Besides, N-processes have a great effect on transferring energy between different phonon modes, thus preventing large deviation from the equilibrium distribution. This redistribution process can pass on the momentum to higher frequency phonons that are strongly influenced by U-processes and impurity scatterings (Tritt, 2004), which lead to the domination of Umklapp scatterings at elevated temperatures.

### Schemes to improve figure-of-merit

The figure-of-merit of a compound can be improved by forming a solid solution with another compound of a similar electronic valence structure but with a different atomic mass, as proposed by Wood (1988). However, the local distortion produced by the introduction of a foreign atom with similar valence does not noticeably scatter free charge carriers but strongly scatters phonons. Hence, the thermal conductivity can be reduced without affecting the electrical transport. This route is demonstrated to be a very efficient approach for all thermoelectric materials, especially in Mg_2_Si_1 − *x*_Sn_*x*_ (Zaitsev et al., 2006). Here, the electrical properties are also enhanced along with the reduction of thermal conductivity, due to the enhancement in the density of states (DOS) in the conduction band of the Sn-substituted Mg_2_Si, while the electron mobility remains the same. The reduction in lattice thermal conductivity directly improves the thermoelectric efficiency, *ZT* and also allows re-optimization of the carrier concentration for additional *ZT* improvement. The strategy for reducing κ_*L*_ can be divided into three schemes (Wan et al., 2010): disorder or distortion of unit cells, resonant scattering by localized rattling atoms and creation of a high density of interfaces. Firstly, the scattering of phonons within a unit cell can be created by rattling structures or point defects such as interstitials, vacancies or by alloying (Rowe, 1995). The second strategy is to use complex crystal structures to separate the electron-crystal from the phonon-glass. Here, the goal is to achieve a phonon glass without disturbing the crystallinity of the electronic region. The third strategy is to scatter phonons at interfaces, leading to the use of multiphase composites mixed on the nanometer scale (Dresselhaus et al., 2007). On the other hand, theoretical predictions suggested that the thermoelectric efficiency could be greatly enhanced by quantum confinement of the electron charge carriers (Hicks and Dresselhaus, 1993). The electron energy bands in a quantum confined structures are progressively narrower as the confinement increases, and the dimensionality decreases. These narrow bands should produce high effective masses, which could leads to large Seebeck coefficients. However, a high-*ZT* device based on this principle has yet to be tested practically.

Based on the above mentioned developments, we have recently employed the modification in electronic band structure as well as the phonon-point-defect scattering via different dopant on the SrSi_2_ alloy to improve its thermoelectric properties (Lue et al., 2009, 2013a,b; Kuo et al., 2012). Efforts on the substitution of third elements in SrSi_2_ such as Al and Ge doping at Si sites, and Ca, Ba and Y doping at Sr sites have been found to be quite effective for the improvement of the thermoelectric performance of SrSi_2_. It is shown in this article that the SrSi_2_ system represents a good opportunity for improving its *ZT*, making this system attractive for possible candidates in the development of highly efficient thermoelectrics.

## SrSi_2_-based alloys

Alkaline-earth-metal disilicide, SrSi_2_ is composed of non-toxic and naturally abundant elements which makes it an eco-friendly material. The SrSi_2_-based silicides with semiconducting or semimetallic behavior tend to draw significant interest in the field of thermoelectrics and photoelectronics (Tritt et al., 1997; Kuo et al., 2005; Lue et al., 2009, 2013a,b). SrSi_2_ has been reported to be a narrow-gap semiconductor with a band gap of 35 meV based on the electrical transport study (Imai et al., 2005), whereas band structure calculations predicted the presence of a sharp pseudogap of about 3 meV (Imai and Watanabe, 2006). However, the value of band gap was estimated to be ~13 meV from our recent study on the electrical transport in SrSi_2_ (Lue et al., 2013a). This value is about four times higher than that of the theoretical predicted value (Imai and Watanabe, 2006), but is smaller as compared with the reported experimental value (Imai et al., 2005). Generally, the materials with sharp electronic band features of a few tens of meV from the Fermi level could be promising candidates for efficient thermoelectrics, according to the model proposed by Mahan and Sofo (1996). In fact, SrSi_2_ was reported to have a large Seebeck coefficient of about 130 μV/K at room temperature. At the same time, this alloy also possesses a relatively high electrical resistivity (~1 mΩ-cm) and thermal conductivity (~5 W/m-K), which hinders its thermoelectric performance (Hashimoto et al., 2007). In order to achieve a better thermoelectric performance, the reduction in the electrical resistivity and thermal conductivity with the enhancement of the Seebeck coefficient in SrSi_2_ has to be realized. To attain these goals, the combination of three factors have been employed in our recent studies on the SrSi_2_-based alloys: (Lue et al., 2009, 2013a,b; Kuo et al., 2012) (a) the reduction of the electrical resistivity through the introduction of charge carriers, (b) the enhancement of the Seebeck coefficient via the band engineering, and (c) the reduction of the thermal conductivity through the introduction of point defects or lattice imperfections.

Polycrystalline SrSi_2_-based alloys can be easily synthesized using a conventional arc-melting technique. Mixture of high-purity elemental metals of the corresponding samples are placed in a water-cooled copper crucible and then melted several times in an argon arc-melting furnace. The weight loss during melting is less than 1% for all compounds. To promote homogeneity, the ingots (~50 g) of the samples are annealed in a vacuum-sealed quartz tube at 800°C for 3 days, and followed by the furnace cooling. Structural study on these SrSi_2_-based alloys was carried out using x-ray diffraction with Cu *K*α radiation, which revealed the cubic crystal structure with space group P4_3_32 (Lue et al., 2009, 2013a,b; Kuo et al., 2012). From x-ray diffraction analysis, it is found that the solubility limit for Al and Ge substitution into Si sites of SrSi_2_ alloy is about 6 at %, while the substitution of Y, Ca, and Ba into Sr sites of SrSi_2_ has a solubility limit of about 10 at %. Above the solubility limit, the peaks of other minor impurity phase start to appear in the spectrum. However, such a minor impurity phase is not likely to affect the thermoelectric properties of the studied SrSi_2_ system. All samples were cut into a rectangular parallelepiped shape with size of about ~1.5 × 1.0 × 6.0 mm^3^ for the transport measurements. Electrical resistivity of these SrSi_2_-based alloys was measured in the temperature range of 10–300 K using a standard four-probe method during the heating cycle. For the electrical resistivity measurement, four indium pads were deposited on the samples by thermal evoparation, on which copper wire contacts were made using silver paint. It consists of four-probe arranged linearly in a straight line at nearly equal distance (~2 mm) between each other. A constant current (1 mA) was applied to the two outer probes, and the voltage across the two inner probes was measured using Keithley 2182 nanovoltmeter. Measurement of the Seebeck coefficients on these alloys was carried out using dc pulse technique in a closed-cycle refrigerator. Seebeck voltages were detected at the junctions of differential thermocouple using a pair of thin Cu wires which are electrically connected to the sample with silver paint at the same positions. The stray thermal emfs were eliminated by applying long current pulses (~100 s) to a chip resistor which served as a heater, where the pulses appeared in an off-on-off sequence. Thermal conductivity of these alloys was measured in the temperature range of 10–300 K using a direct heat-pulse technique. For the thermal conductivity measurement, one end of the sample was glued (with thermal epoxy) to a copper block that served as a heat sink, while a calibrated chip resistor (100 Ω at room-temperature) as a heat source was connected to the other end. The temperature difference was detected by using an E-type differential thermocouple with junctions thermally attached to two well-separated positions along the sample. The temperature difference between junctions was kept at about ~0.5 K to minimize the heat loss via thermal radiation, and the sample space was also maintained in a good vacuum (~10^−4^ torr) during measurements to avoid heat loss through convection. In the present article, we attempt to understand the doping effects on the thermoelectric properties of the SrSi_2_-based alloys, and also try to reveal suitable alternative routes for the further improvement of the thermoelectric performance in the intermetallic SrSi_2_ system.

### SrSi_2−*x*_Al_*x*_: hole doping with negative chemical pressure

The dopant aluminum (Al) has one less valence electron and lager atomic radius than that of the host atom Si (Table 1). Hence, the Al doping is expected to induce the holes as extra charge carriers and negative chemical pressure in the lattice of SrSi_2_. The combination of these effects could greatly influence the electrical conductivity and band structure of the SrSi_2 − *x*_Al_*x*_ alloys. From the x-ray diffraction study on these samples, it is found that the lattice constant increases with increasing Al concentration (*x*), due to the induced negative chemical pressure by substitution of Al into the Si sites of SrSi_2_ (Kuo et al., 2012).

**Table 1:** **The values of atomic radius and atomic mass of substituent and host elements in both Sr-site and Si-site of SrSi_2_ alloy**.

**A-site element**	**Atomic radius (Å)**	**Atomic mass (g/mol)**	**B-site element**	**Atomic radius (Å)**	**Atomic mass (g/mol)**
Sr (host)	2.15	87.62	Si (host)	1.11	28.09
Ba	2.22	137.33	Ge	1.22	72.63
Ca	1.97	40.08	Al	1.43	26.98
Y	1.80	88.91	–	–	–

**Figure 1:**
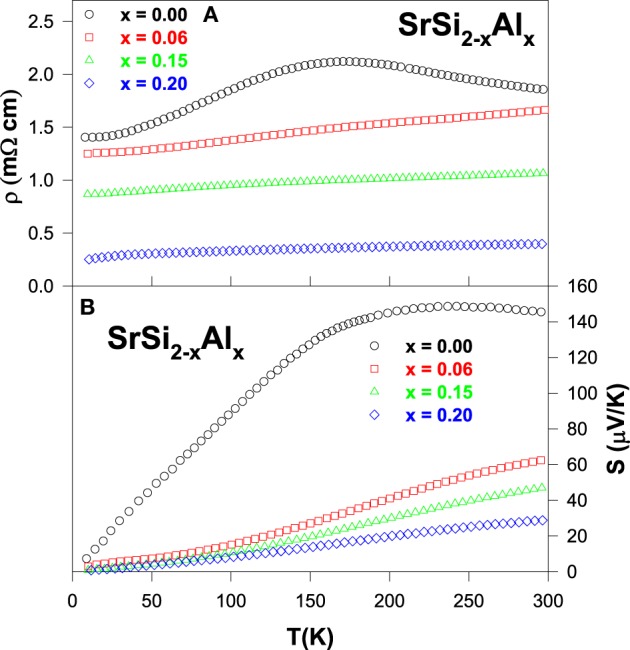
**(A)** Electrical resistivity and **(B)** Seebeck coefficient with temperature for the SrSi_2 − *x*_Al_*x*_ alloys.

Electrical resistivity and Seebeck coefficient as a function of temperature for SrSi_2 − *x*_Al_*x*_ samples are illustrated in Figures 1A,B, respectively. The pure SrSi_2_ alloy shows a semimetallic feature with a relatively large resistivity of about 1 mΩ cm. The magnitude of ρ is found to increase slightly with rising temperature, tends to saturate around 170 K, and then starts decreasing upon further heating. Such a behavior is associated to the low DOS at the Fermi level (*E_F_*). Upon Al doping, the resistivity is found to reduce gradually in the entire temperature range (Figure 1A), as a consequence of the increased carrier density of holes, which results in a metallic character for the Al-substituted SrSi_2_ alloys (Kuo et al., 2012). On the other hand, the narrowing of band gap in these substituted alloys is expected, due to the induced chemical pressure by the Al substitution which could also leads to a better metallic behavior in their electrical transport property (Figure 1A).

The sign of the Seebeck coefficients of SrSi_2 − *x*_Al_*x*_ systems is positive in the temperature range of 10–300 K, suggesting that the hole-type carriers dominate the thermoelectric transport in these alloys (Figure 1B). Upon cooling, the Seebeck coefficient of the pure SrSi_2_ alloy increases slowly with temperature up to 250 K and then decreases rapidly with temperature below 150 K. The broad hump in *S* near 250 K is presumably due to the thermally excited quasi-particles across the narrow pseudogap at high temperatures (Lue et al., 2013a). On the other hand, the Al substitution in SrSi_2_ leads to a significant reduction in *S* over entire temperature range (Figure 1B). However, it is found that the *S* value increases quite linearly with increasing temperature above 200 K, indicating the diffusive nature of the thermoelectric transport. The deduced value of Fermi energy (*E_F_*) is found to be enhanced with Al content, indicative of the enhancement of the Fermi energy and/or slight shift in the position of *E_F_* (Kuo et al., 2012). For ordinary metals, the Seebeck coefficient can be described by well-known Mott formula (Mott and Jones, 1936), and the low-temperature *S* can be expressed as

(3) $$S\propto {\left(\frac{1}{N(E)}\frac{\partial N(E)}{\partial E}\right)}_{E\;=\;E_{F}},$$

based on a one-band model with an energy-independent relaxation time, where *N*(*E*) is the electronic DOS. According to Equation (3), the variation in the Seebeck coefficient can be associated with the change of the Fermi level DOS. Since, the magnitude of *S* is proportional to the slope of the DOS around *E_F_*, a slight shift in the position of *E_F_* could cause a considerable change in the value of *S*. Hence, the change in electronic band structure near the Fermi energy clearly modifies the Seebeck coefficients of SrSi_2 − *x*_Al_*x*_ as increasing Al content (see Figure 1B).

**Figure 2:**
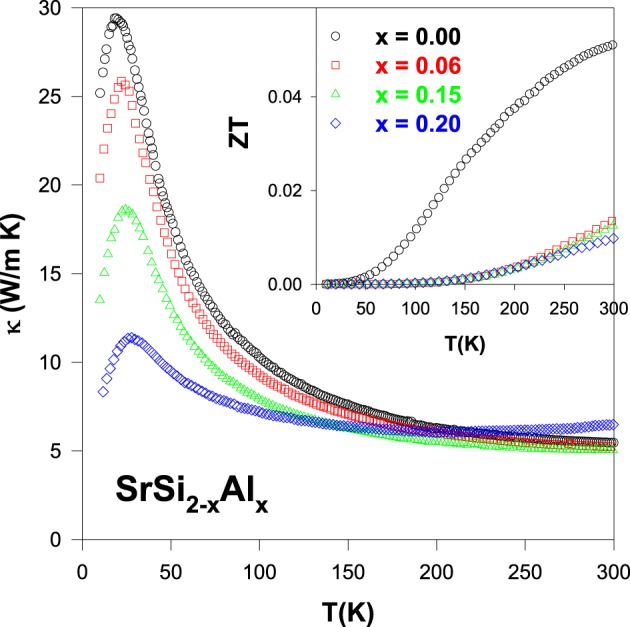
**Measured thermal conductivity (κ) of the SrSi_2 − *x*_Al_*x*_ alloys**. Inset shows figure-of-merit, *ZT* as a function of temperature for the SrSi_2 − *x*_Al_*x*_ systems.

Figure 2 shows the measured thermal conductivity as a function of temperature for SrSi_2 − *x*_Al_*x*_. A gradual increase in κ with decreasing temperature, followed by a distinct maximum (phonon peak) near 25 K and then a steep fall below 25 K, is observed for pure SrSi_2_. This is a typical behavior of solids, and the peak appears at the temperature where the phonon mean free path is approximately equal to the crystal site distance, ascribed to the Umklapp process. The room-temperature κ value for the Al-substituted SrSi_2_ alloys lies between 5.5 and 6.5 W/m-K, weakly affected by the composition of Si/Al. However, the magnitude and the characteristics of thermal conductivity at low temperatures reduce substantially with Al content (Kuo et al., 2012), mainly due to the mass fluctuations between Si and Al, since their atomic size difference is very high (see Table 1). The observed variation in low-temperature thermal conductivity will be further explored in detail in the Section Fitted Lattice Thermal Conductivity Data of the Optimized SrSi_2_-Based Alloys Using Debye Equation based on the framework of the Debye model.

The dimensionless figure-of-merit (*ZT*) as a function of temperature of the SrSi_2 − *x*_Al_*x*_ alloys is plotted in the inset of Figure 2. It is evident that a considerable reduction in the *ZT* value for the Al-substituted alloys has been observed, as compared to pure SrSi_2_. The room-temperature values of ρ, *S*, κ, power factor (*PF*) and *ZT* of the pure SrSi_2_ and SrSi_1.94_Al_0.06_ alloys are listed in Table 2. It can be found that the huge drop in *ZT* value is mainly due to the reduction in the power factor, as the magnitude of *S* decreases by a factor of two while the magnitude of ρ decreases slightly for the SrSi_1.94_Al_0.06_ alloy (see Figures 1, 2). Such a finding indicates that tuning the carrier concentration (or Fermi level DOS) for the purpose of enhancing power factor of a particular alloy has to be conducted carefully.

**Table 2:** **The deduced values of resistivity, Seebeck coefficient, thermal conductivity, thermoelectric power factor, and figure-of-merit of SrSi_2_-based alloys at room-temperature**.

**Sample name**	**ρ (mΩ cm)**	***S* (μV/K)**	**κ (W/m-K)**	**κ_*L*_ (W/m-K)**	***PF* (10^−3^ W/m-K^2^)**	***ZT***
SrSi_2_	1.85	131.4	5.46	5.10	0.9	0.051
SrSi_1.94_Al_0.06_	1.66	63.1	5.23	4.79	0.2	0.01
SrSi_1.94_Ge_0.06_	1.29	144.3	5.10	4.28	1.6	0.13
Sr_0.9_Ca_0.1_Si_2_	1.83	220.8	5.17	5.18	2.7	0.17
Sr_0.93_Ba_0.07_Si_2_	1.42	156.7	4.90	4.90	1.7	0.11
Sr_0.92_Y_0.08_Si_2_	0.28	134.3	4.88	4.51	6.4	0.41
Sr_0.94_Si_2_	3.30	168.1	4.86	4.63	0.9	0.053
Sr_1.06_Si_2_	13.4	88.8	3.91	3.86	0.1	0.01
Sr_0.77_Ca_0.1_Si_2_	1.39	143.9	1.66	1.13	1.5	0.27

### SrSi_2−*x*_Ge_*x*_: negative chemical pressure

Since the combined changes in carrier concentration and band gap of SrSi_2_ by Al doping is failed to improve its *ZT* value (see Section SrSi_2 − *x*_Al_*x*_: Hole Doping With Negative Chemical Pressure), we subsequently conducted the substitution of germanium (Ge) for Si which has the same valence as Si but with a larger atomic radius and atomic mass (Table 1). Here, the strategy is to induce a negative chemical pressure in the SrSi_2_ lattice without affecting its carrier concentration. The X-ray diffraction result shows that the lattice constant gradually increases with increasing the Ge content, suggesting that the Si atoms are successfully replaced by Ge atoms in these alloys, according to Vegard's law (Lue et al., 2013a).

**Figure 3:**
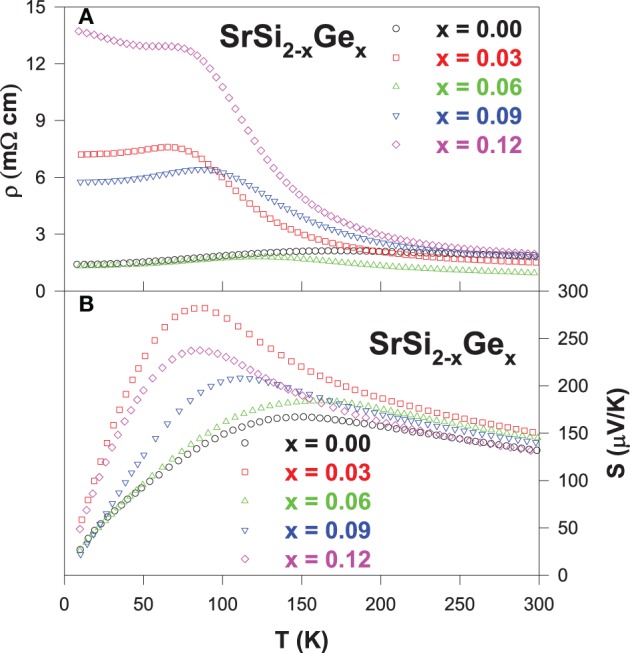
**Temperature-dependent electrical resistivity (A), and Seebeck coefficient (B) of the SrSi_2 − *x*_Ge_*x*_ alloys**.

The measured electrical resistivity and Seebeck coefficient of (0.00 ≤ *x* ≤ 0.12) are shown in Figures 3A,B, respectively. It is clear that the semiconducting feature becomes more pronounced in the Ge-substituted alloys, as evidenced by the increase in the negative temperature coefficient of resistivity (TCR) as well as the extension of the TCR behavior down to a lower temperature of about ~70 K for SrSi_1.97_Ge_0.03_. We estimated the band gap value (*E_g_*) of the Ge-substituted SrSi_2_ alloys by fitting the high-temperature resistivity data to the Arrhenius equation, ρ = ρ_0_exp(*E_g_*/2*k_B_T*), where *k_B_* is the Boltzmann constant. The band gap value of pure SrSi_2_ is estimated to be about 13 meV, which increases with Ge content to the value of ~51 meV for SrSi_1.88_Ge_0.12_ (Lue et al., 2013a). However, all these Ge-substituted alloys still exhibit semimetallic behavior with a downturn feature at low temperatures (Figure 3A).

From the Seebeck coefficient data (Figure 3B), it is found that the room-temperature *S* value increases marginally from 130 μV/K (SrSi_2_) to 150 μV/K for SrSi_1.97_Ge_0.03_ alloy, and then decreases for further Ge substitution. In addition, a considerable enhancement in the *S* value at low temperatures is clearly observed with Ge substitution, with the highest value of about 280 μV/K near 85 K for the alloy with *x* = 0.03. However, it is noted that the *S* values of exhibit no systematic variation with *x*, indicating that the observed composition dependence in *S* may not be linked to the changes in the mobility or carrier concentration (Lue et al., 2013a), since Ge is isoelectronic to Si. Besides, the carrier scattering relaxations are limited by impurity scatterings as evident from the high residual resistivity for the Ge-substituted alloys except for *x* = 0.06, and the phonons have not yet started to play a significant role at low temperatures. From these observations, the electronic diffusion contribution to the Seebeck coefficient can be simply related to Mott's equation (Mott and Jones, 1936),

(4) $$S=\frac{\pi^{2}k_{B}^{2}}{2eE_{F}}T=bT,$$

From Equation (4), it is found that the *S* value of pure SrSi_2_ varies quite linearly with temperature up to 150 K, and then starts to deviates from the linear behavior above 150 K. On the other hand, the linear temperature dependence of *S* for the Ge-substituted alloysis limited to much lower temperatures below 100 K. Such an observation suggests that the metallic diffusive contribution to the measured *S* of the Ge-substituted alloys is reduced gradually with Ge content, revealing a shift in the electronic band structure toward semiconducting-like behavior in these Ge-substituted alloys. From the linear fitting of the low-temperature Seebeck coefficient to the Equation (4), the estimated *b* value is found to be large for all substituted alloys as compared to that of pure SrSi_2_ (Lue et al., 2013a). Since the parameter *b* is inversely proportional to the Fermi energy *E_F_*, the increase in the *b* value with Ge content indicates that the slight shift in the position of *E_F_*, which could in turn alter the DOS near the Fermi level. The observed band gap broadening and the altering of DOS near the Fermi level subsequently modify the electrical resistivity and Seebeck coefficient characteristics of the Ge-substituted SrSi_2_ alloys (see Figures 3A,B) (Imai et al., 2007).

**Figure 4:**
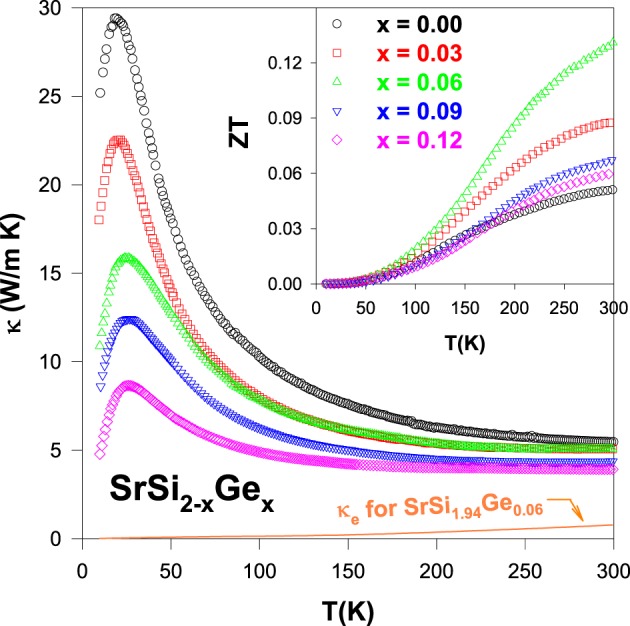
**Total thermal conductivity (κ) as a function of temperature for the SrSi_2 − *x*_Ge_*x*_ alloys and κ_*e*_ for SrSi_1.94_Ge_0.06_ alloy**. Inset shows temperature-dependent *ZT* value of the SrSi_2 − *x*_Ge_*x*_ alloys.

The temperature dependence of thermal conductivity of the alloys is displayed in Figure 4. The room-temperature thermal conductivity (κ_*RT*_) of the Ge-substituted alloys is found to vary from 5.1 W/m-K (SrSi_2_) to 3.6 W/m-K (SrSi_1.88_Ge_0.12_), representing a moderate dependence of κ_*RT*_ with the composition of Si/Ge. However, it is noticed that the low-temperature phonon peak in κ of the Ge-substituted alloys is suppressed significantly with increasing Ge content, indicating a strong enhancement in the phonon scattering by Ge substitution (Lue et al., 2013a). Besides, the estimated electronic thermal conductivity, κ_*e*_(*T*) of the SrSi_1.94_Ge_0.06_ using the Wiedemann–Franz law is shown as dotted lines in the Figure 4, which has the highest κ_*e*_ among the Ge-substituted alloys. From this calculation, it is evident that the total thermal conductivity of the Ge-substituted alloys is mainly due to the lattice phonons rather than the charge carriers.

The thermoelectric performance, *ZT* vs. *T* of the Ge-substituted SrSi_2_ alloys is displayed in the inset of Figure 4. It is apparent that a substantial enhancement in the *ZT* value for the Ge-substituted alloys compared to that of pure SrSi_2_. The maximum room-temperature *ZT* value of about 0.13 for SrSi_1.94_Ge_0.06_ alloy is obtained, almost three times larger than that of SrSi_2_ (Table 2). Even though such a *ZT* value is still small compared to that of the optimized Bi_2_Te_3_ (Rowe, 1995). The thermoelectric power factor (*S*^2^/ρ) of SrSi_1.94_Ge_0.06_ is estimated to be about 1.6 × 10^−3^ W/m-K^2^ (Table 2), comparable to the thermoelectric materials such as Bi_2_Te_3_, K_2_Bi_8_Se_13_, and CoSi_1 − *x*_Ge_*x*_ (Rowe, 1995; Chung et al., 1997; Skoug et al., 2009). Hence, it is obvious that the thermoelectric power factor of SrSi_2_ can be effectively enhanced by band engineering through the introduction of negative chemical pressure in the SrSi_2_ lattice with Ge substitution.

### Sr_1 − *x*_Ca_*x*_Si_2_ and Sr_1−*x*_Ba_*x*_Si_2_: positive and negative chemical pressure

Based on the encouraging observation from Ge substitution onto the Si sites of SrSi_2_ alloy in the Section SrSi_2−_*_*x*_* Ge_*x*_: Negative Chemical Pressure, an investigation of thermoelectric properties on Calcium (Ca) and Barium (Ba) substituted SrSi_2_ alloys, namely Sr_1 − *x*_Ca_*x*_Si_2_ and Sr_1 − *x*_Ba_*x*_Si_2_, is in order. Here, the substitution of a smaller atomic size Ca could introduce a positive chemical pressure in the SrSi_2_ lattice (Table 1). On the other hand, the replacement of Sr with a larger atomic size of Ba could cause an expansion of the lattice (Table 1), equivalent to the production of a negative chemical pressure in the system. As expected, the x-ray diffraction results show that the lattice constant decreases with increasing Ca content, while the addition of Ba leads to a gradually increase in the lattice constant (Lue et al., 2013b).

**Figure 5:**
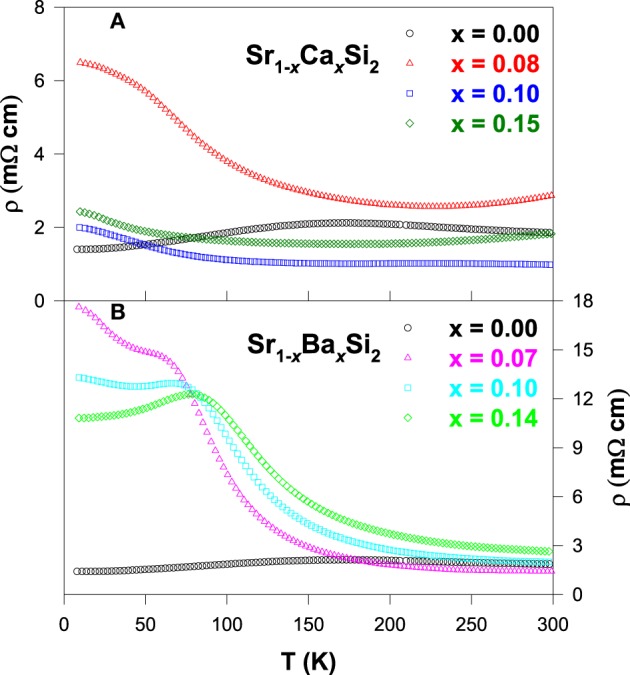
**Temperature-dependent electrical resistivity of (A) Sr_1 − *x*_Ca_*x*_Si_2_ and (B) Sr_1 − *x*_Ba_*x*_Si_2_ alloys**.

Figure 5 illustrates the temperature dependence of the electrical resistivity for the Sr_1 − *x*_Ca_*x*_Si_2_ and Sr_1 − *x*_Ba_*x*_Si_2_ alloys. As seen in Figure 5A, the substitution of Ca in SrSi_2_ has an effect of lowering the magnitude of ρ(*T*) in the intermediate temperature range. It is also noted that ρ(*T*) exhibits a positive TCR above 200 K in these Sr_1 − *x*_Ca_*x*_Si_2_ alloys (Lue et al., 2013b). On the other hand, Ba substitution (Sr_1 − *x*_Ba_*x*_Si_2_) results in a strong semiconducting characteristics with a negative TCR above 90 K (Figure 5B), while a slight reduction in ρ(*T*) is noticed near room temperature. These observations indicate that the pressure effect indeed plays a critical role on the band characteristics of SrSi_2_ (Imai et al., 2011), as predicted by the band structure calculations (Imai and Watanabe, 2006). Besides, the residual resistivity of both Ca and Ba substituted alloys increases with the substitution up to *x* ~ 0.08 and then decreases slightly with further addition of Ca and Ba, implying an enhancement of impurity scattering in these substituted alloys.

**Figure 6:**
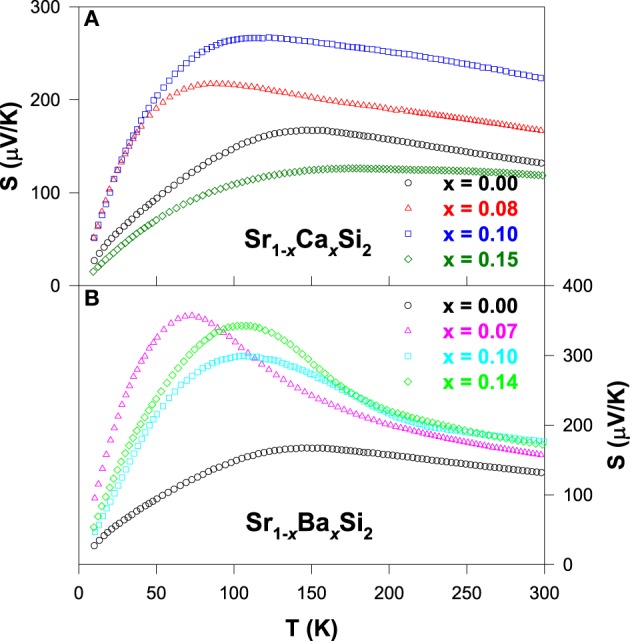
**Seebeck coefficients as a function of temperature for (A) Sr_1 − *x*_Ca_*x*_Si_2_ and (B) Sr_1 − *x*_Ba_*x*_Si_2_ alloys**.

For the Ca and Ba substituted alloys, the Seebeck coefficient of each composition develops a broad maximum below about 200 K as seen in Figure 6, presumably due to the contribution of the thermally excited electrons across the gap or pseudogap (Lue and Kuo, 2002). It is noticed that the room-temperature *S* value increases gradually with the substitution up to *x* ~ 1.0 and then decreases with further substitution of Ca and Ba. The maximum room-temperature *S* value is achieved with the optimum substitution level of *x* ~ 0.1 for both Sr_1 − *x*_Ca_*x*_Si_2_ and Sr_1 − *x*_Ba_*x*_Si_2_ cases (Lue et al., 2013b). In addition, the linear behavior in the *S*(*T*) below 100 K shifts to low temperatures, signifying that the metallic diffusion nature in *S* of these substituted alloys gradually diminishes, especially for the Ba-substituted alloys (Figure 6B). As a result, the semiconducting-like feature prevails in a wide range of temperature, consistent with their electrical resistivity data (Figure 5).

**Figure 7:**
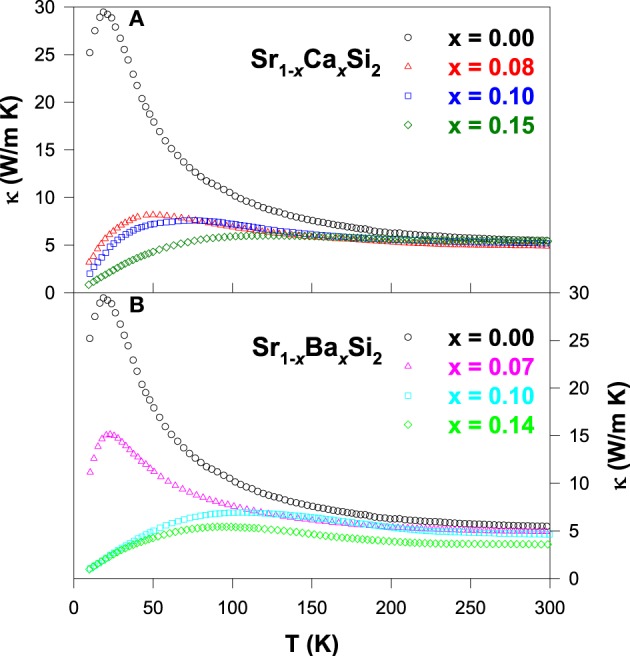
**Measured thermal conductivity vs. temperature for (A) Sr_1 − *x*_Ca_*x*_Si_2_ and (B) Sr_1 − *x*_Ba_*x*_Si_2_ alloys**.

In Figure 7, the thermal conductivity for all Ca and Ba substituted alloys exhibits a weak temperature variation above 200 K, with a robust room-temperature κ value of about ~5 W/m-K. However, the low-temperature phonon peak is suppressed significantly with increasing Ca and Ba contents, indicating the strong enhancement in the scattering of phonon by lattice defects induced by the substitution. This huge drop in the κ value of the substituted alloys is mainly attributed to the phonon-point-defect scattering, which arises from the effect of mass fluctuation between host atom (Sr) and the dopant (Ca and Ba) due to the difference in their atomic mass and radius (Table 1). From the estimation of κ_*e*_(*T*), it is found that the measured thermal conductivity is essentially associated with the phonons (Lue et al., 2013b), similar to other substituted SrSi_2_ alloys discussed in this article.

**Figure 8:**
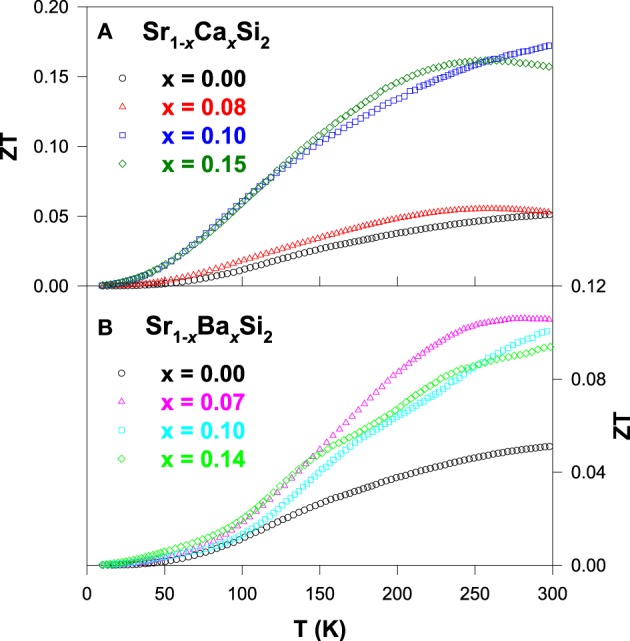
**The *ZT* value vs. temperature for (A) Sr_1 − *x*_Ca_*x*_Si_2_ and (B) Sr_1 − *x*_Ba_*x*_Si_2_ alloys**.

Figure 8 shows the *ZT* value as a function of temperature for the Sr_1 − *x*_Ca_*x*_Si_2_ and Sr_1 − *x*_Ba_*x*_Si_2_ alloys. It is clear that a significant enhancement in the *ZT* value is achieved for both Ca and Ba substituted SrSi_2_ alloys over the temperature range of 10–300 K. The maximum room-temperature *ZT* values of about 0.17 and 0.11 were obtained for Sr_0.9_Ca_0.1_Si_2_ and Sr_0.93_Ba_0.07_Si_2_, respectively (Table 2), mainly due to the enhancement in their Seebeck coefficients (Lue et al., 2013b). However, the high *ZT* value of 0.17 for the Sr_0.9_Ca_0.1_Si_2_ sample at room temperature is still smaller than that of the optimized Bi_2_Te_3_ (Rowe, 1995). Yet, we have clearly demonstrated that the thermoelectric performance can be considerably improved by the introduction of chemical pressure through the substitution of Ca and Ba in the SrSi_2_ system.

### Sr_1 − *x*_Y_*x*_Si_2_: electron doping with positive chemical pressure

In order to explore the thermoelectric properties with respect to the additional carrier concentration in the SrSi_2_, we have studied the effect of electron doping via yttrium (Y) substitution on the strontium (Sr) sites. From the x-ray diffraction analysis on the Sr_1 − *x*_Y_*x*_Si_2_ alloys, it is observed that the lattice constant decreases gradually with increasing Y content (Lue et al., 2009), due to a positive chemical pressure induced by Y substitution since Y has a smaller atomic size than that of Sr (Table 1). In addition, Y has one more electron in its valence shell than that of Sr, which could cause modifications in the band structure of SrSi_2_, which will in turn have a significant influence on the thermoelectric properties of these alloys.

**Figure 9:**
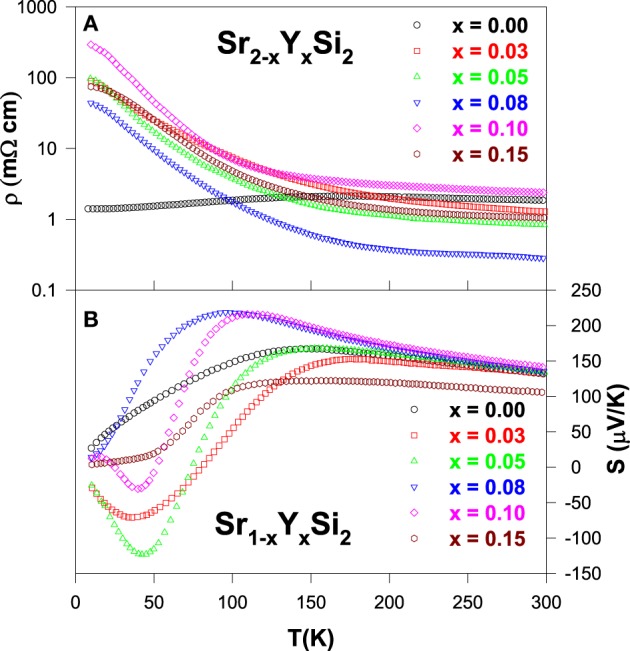
**(A)** Electrical resistivity and **(B)** Seebeck coefficient data of the Sr_1 − *x*_Y_*x*_Si_2_ alloys.

From the electrical resistivity data (Figure 9A), all Y-substituted alloys show semiconducting behavior with a negative TCR, contrary to the semimetallic nature of pure SrSi_2_. However, the fitting of resistivity data to the variable range hopping mechanism reveals that the Y-substituted alloys are semimetals with pseudogaps in the vicinity of the Fermi levels (Lue et al., 2009). In fact, along with the chemical pressure induced by Y substitution in the SrSi_2_ lattice, the employment of Y as an electron donor could modify its electronic structure, which is responsible for the observed behavior in the resistivity data of Y-substituted alloys (Figure 9A).

For the Y substitution level of *x* = 0.03, the Seebeck coefficient appears to be smaller than that of pure SrSi_2_ ascribed to the band filling effect (Figure 9B), as Y has one more valence electron than Sr (Lue et al., 2009). Upon further substitution (*x* ≥ 0.05), the *S* value increases gradually with the Y content up to *x* = 0.08 and then decreases for *x* > 0.08. The variation in the *S*(*T*) characteristics of these alloys, especially the *S* value being negative at low temperatures below 100 K (Figure 9B), indicating a significant change in the electronic band structure as a result of electron doping to an originally *p*-type SrSi_2_ system. In particular, the negative peaks observed in the *S*(*T*) data with *x* = 0.03, 0.05, and 0.10 are presumably due to the two-carrier conduction mechanism (Lue et al., 2009), which can be described as

(5) $$S=\frac{\sigma_{n}}{\sigma_{n}+\sigma_{p}}S_{n}+\frac{\sigma_{p}}{\sigma_{n}+\sigma_{p}}S_{p},$$

where, *S*_*n*,*p*_ and σ _*n*,*p*_ are the Seebeck coefficients and the electrical conductivities for *n*- (electron) and *p*-type (hole) charge carriers, respectively. Due to the opposite sign for *S_n_* and *S_p_*, the observed sign of *S* depends on the dominance of the *n*- and *p*-type charge carriers. From this observation (Figure 9B), it is evident that the Y-substituted alloys have dominant *n*-type carriers at low temperatures via electron doping (Lue et al., 2009). On the other hand, the *p*-type carriers govern the thermal transport at elevated temperatures above 100 K, presumably due to the relatively higher mobility for holes than that of electrons.

**Figure 10:**
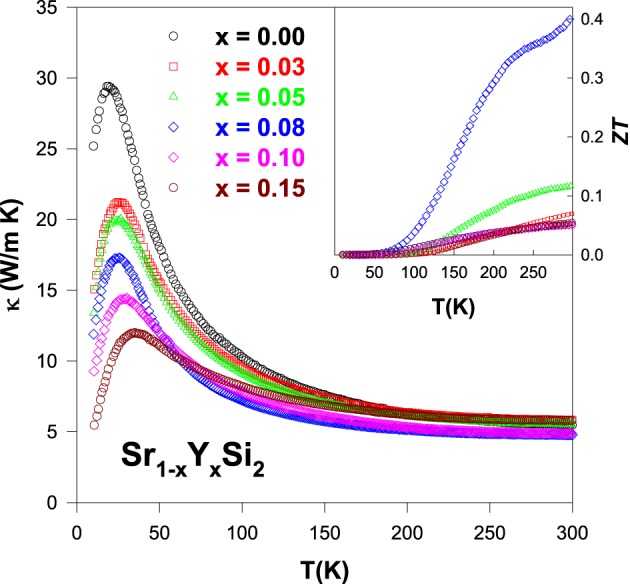
**Measured thermal conductivity (κ) of the Sr_1 − *x*_Y_*x*_Si_2_ alloys over the temperature range of 10–300 K**. Inset shows the plot of the *ZT* value vs. *T* for the Sr_1 − *x*_Y_*x*_Si_2_ alloys.

Substitution of Y onto Sr sites of SrSi_2_ alloy shows a negligible reduction in high-temperature thermal conductivity above 200 K (Figure 10), since the mass difference between Y and Sr is extremely small (Table 1). However, the low-temperature phonon peak in κ(*T*) below 50 K appears to be reduced strongly with the substitution of Y, largely due to the enhancement of phonon-point-defect scattering (Lue et al., 2009). Moreover, the estimation of the electronic thermal conductivity indicates that the phonon thermal transport dominates the total thermal conductivity, similar to other substituted SrSi_2_ alloys.

The calculated *ZT* value for the Sr_1 − *x*_Y_*x*_Si_2_ alloys using the data of ρ, *S*, and κ over the temperature range of 10–300 K are plotted in the inset of Figure 10. The highest *ZT* value of 0.41 is achieved for the Sr_0.92_Y_0.08_Si_2_ alloy at room temperature, the highest one among the all studied SrSi_2_-based alloys presented here. It is due to the fact that the power factor of this alloy, Sr_0.92_Y_0.08_Si_2_ is very high (6.4 × 10^−3^ W/m-K^2^) compared to that of the other substituted SrSi_2_ alloys (Table 2), which is a result of the effective optimization of ρ and *S* values by Y substitution via the modification in electronic band structure and carrier mobility (Lue et al., 2009; Chen et al., 2013). However, the lattice thermal conductivity of the optimized Sr_0.92_Y_0.08_Si_2_ system is still high near room temperature, which needs to be reduced by other routes in order to further improve its *ZT* value.

### Sr_*y*_Si_2_ and Sr_0.9 − δ_Ca_0.1_Si_2_: Sr-deficiency

#### Sr_*y*_Si_2_

With the motivation to further reduce lattice thermal conductivity in the SrSi_2_ system, the Sr-deficient compounds Sr_*y*_Si_2_ with *y* = 0.94 and 0.99 and the Sr-rich compound *y* = 1.06 are prepared. Here, the concept of Sr-deficiency is to create lattice imperfections to reduce the lattice thermal conductivity without introducing extra carriers to the SrSi_2_ system.

**Figure 11:**
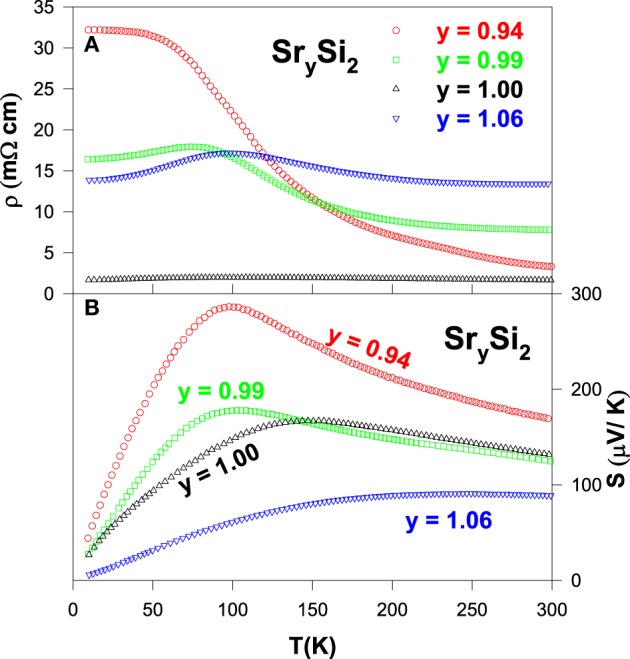
**(A)** Electrical resistivity and **(B)** Seebeck coefficient of the Sr_*x*_Si_2_ alloys over the temperature range of 10–300 K.

The measured electrical resistivity and Seebeck coefficient data on the Sr_*y*_Si_2_ compounds are displayed in Figures 11A,B, respectively. It is noticed that the Sr-deficiency has resulted in a significant reduction in the value of ρ over entire temperature range for *y* = 0.99, while ρ increases for higher Sr-deficiency level with *y* = 0.94. On the other hand, the Sr-rich compound Sr_1.06_Si_2_ shows an increase in ρ value compared to pure SrSi_2_ above 100 K, whereas the ρ value is smaller below 100 K. Overall, all compounds of Sr_*y*_Si_2_ is semimetallic in nature. Upon the introduction of Sr-deficiency, the measured *S* value is found to increase considerably for the *y* = 0.94 compound. However, the Sr-rich compound (*y* = 1.06) has a lower *S* value than that of pure SrSi_2_ over the entire temperature range.

**Figure 12:**
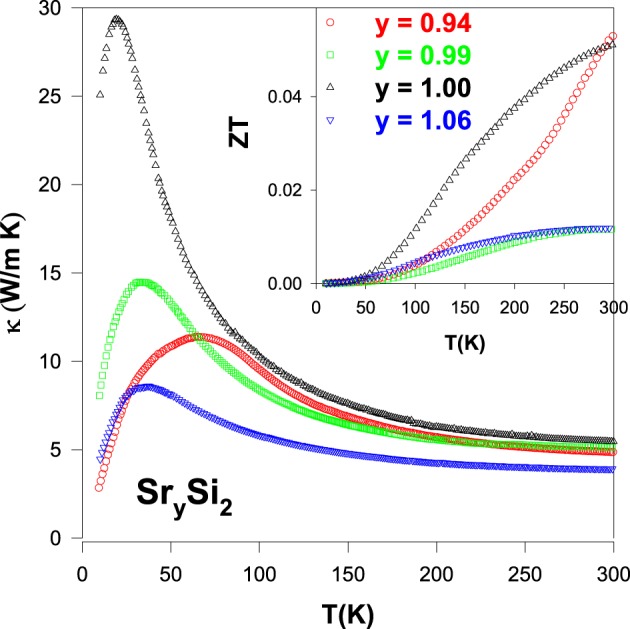
**Temperature dependence of thermal conductivity (κ) of the Sr_*y*_Si_2_ alloys**. Inset shows the *ZT* value vs. temperature for the Sr_*y*_Si_2_.

From Figure 12, it is noted that both Sr-deficient and Sr-rich SrSi_2_ compounds show a significant reduction in the height of the phonon peak with a slight decrease in the κ values above 100 K, as compared to that of the pure SrSi_2_ compound. The evaluated *ZT* value as a function of temperature for these alloys is displayed in the inset of Figure 12. It is worthwhile mentioning that a reduction in the *ZT* value is seen in the Sr-deficient and Sr-rich SrSi_2_ alloys as their power factor reduces, apart from a tiny increment in *ZT* near a room temperature for the Sr_0.94_Si_2_ compound. It is noted that Sr_0.94_Si_2_ exhibits stronger temperature dependence in the *ZT* value with increasing temperature above 250 K. Therefore, it is expected that a higher value of *ZT* is presumably available at elevated temperatures for this alloy. Nevertheless, the present study shows that a small amount of Sr-deficiency (or Sr-rich) in the SrSi_2_ system has only a marginal effect on its overall thermal conductivity near room temperature, and other approach has to be explored.

#### Sr_0.9 − δ_Ca_0.1_Si_2_

In the Section Sr_1 − *x*_Ca_*x*_Si_2_ and Sr_1 − *x*_Ba_*x*_Si_2_: Positive And Negative Chemical Pressure, we have shown the room-temperature Seebeck coefficient of 10% Ca substituted SrSi_2_ (Sr_0.9_Ca_0.1_Si_2_) is about 225 μV/K (Lue et al., 2013b), nearly two times larger than that of the pure SrSi_2_. Such an enhancement in the Seebeck coefficient leads to a considerably high thermoelectric power factor, *S*^2^/ρ of about ~2.7 × 10^−3^ W/m-K^2^ for Sr_0.9_Ca_0.1_Si_2_ system. The challenge which still remains is to reduce the lattice thermal conductivity at high temperatures for further improvement in their thermoelectric performance. Subsequently, we have synthesized Sr-deficient Sr_0.9_Ca_0.1_Si_2_, i.e., Sr_0.9 − δ_Ca_0.1_Si_2_ with δ = 0.00–0.20 to further improve its *ZT* value near room temperature (Lue et al., 2013c).

**Figure 13:**
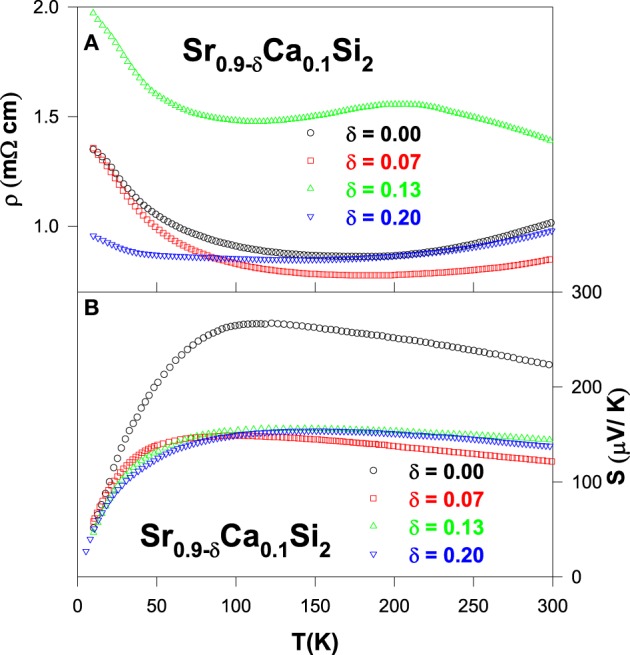
**(A)** Electrical resistivity and **(B)** Seebeck coefficient of the Sr_1 − δ_ Ca_0.1_Si_2_ alloys.

The measured electrical resistivity data of Sr_0.9 − δ_Ca_0.1_Si_2_-based alloys show a non-systematic variation with respect to Sr-deficiency, as shown in Figure 13A. Normally, the increase in the Sr-deficiency is expected to increase the magnitude of ρ due to enhanced disorder scattering. However, the room-temperature resistivity value is found to decrease considerably for the Sr-deficient alloys than that of Sr_0.9_Ca_0.1_Si_2_ (Table 2). The TCR is found to be negative for these alloys at lower temperatures (Lue et al., 2013c), suggesting the strong disorder scattering to the electrical transport in these alloys. At high temperatures, the Sr-deficient Sr_0.77_Ca_0.1_Si_2_ alloy exhibits a semiconducting-like character with a negative TCR above 220 K.

The measured Seebeck coefficients of Sr_0.9 − δ_Ca_0.1_Si_2_ alloys exhibit a broad maximum below 120 K (Figure 13B), ascribed to the contribution of the thermally excited electrons across the pseudogap (Chen and Tian, 2011). The positive sign of *S* for these Sr-deficient alloys indicates that the holes are the dominate carriers for the thermoelectric transport, consistent with a hole pocket in the vicinity of the Fermi level between *G* and *R* points of the electronic structure (Chen and Tian, 2011). Upon introduction of Sr-deficiency into the Sr_0.9_Ca_0.1_Si_2_ system, the value of *S* reduces to less than 150 μV/K from 225 μV/K. Furthermore, the Sr-deficiency is equivalent to the decrease in the number of hole carriers, which could modify the electronic band structure of Sr_0.9_Ca_0.1_Si_2_, which leads to the observed modification in both electrical resistivity and Seebeck coefficients (Lue et al., 2013c). While the decrease in the magnitude of *S* is disadvantageous for achieving high thermoelectric performance, however the moderate drop in *S* could still yield an increase in the *ZT* value with increasing temperature if the lattice thermal conductivity can be significantly reduced with Sr-deficiency.

**Figure 14:**
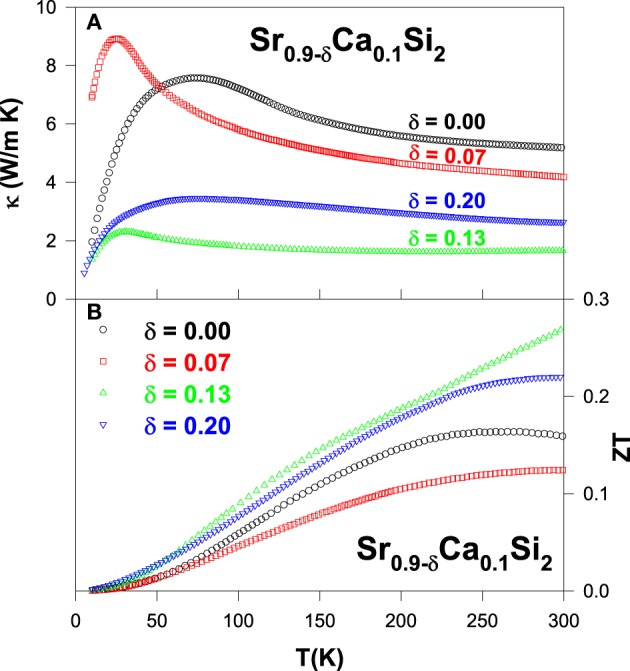
**(A)** Measured thermal conductivity and **(B)** *ZT* value vs. temperature for the Sr_0.9 − δ_ Ca_0.1_Si_2_ alloys.

From the measured thermal conductivity data (Figure 14A), it is seen that the room-temperature κ value drops substantially with increasing the Sr-deficiency in the Sr_0.9_Ca_0.1_Si_2_ alloy. It should be noted that the reduction of κ by substituting third elements in SrSi_2_ has only a marginally effect near room temperature in our earlier studies (Lue et al., 2009, 2013a,b). However, a notably low value of κ = 1.67 W/m-K at room-temperature is achieved for Sr-deficient Sr_0.77_Ca_0.1_Si_2_ alloy, representing a factor of three reduction in κ as compared to the stoichiometric SrSi_2_. This observation clearly demonstrates that the room-temperature thermal conductivity can be effectively suppressed by introducing Sr-deficiency/lattice imperfections in the Sr_0.9_Ca_0.1_Si_2_ system. On the other hand, low-temperature phonon peak in the thermal conductivity of the Sr_0.9_Ca_0.1_Si_2_ alloy has also been significantly diminished with the introduction of Sr-deficiency (see Figure 14A).

The estimated *ZT* value vs. temperature for the Sr_0.9 − δ_Ca_0.1_Si_2_ alloys is illustrated in Figure 14B. A maximum room-temperature *ZT* value of about 0.27 is obtained for the Sr_0.77_Ca_0.1_Si_2_ alloy, almost twice larger than that of Sr_0.9_Ca_0.1_Si_2_ (Table 2). Besides, the *ZT* value increases rapidly with increasing temperature for Sr_0.77_Ca_0.1_Si_2_. A realistic extrapolation of the ρ, *S*, and κ data to high temperatures yields a possible maximum *ZT* value of about 0.5 at around 800 K. This estimated result is mainly associated with the significant suppression in κ via the introduction of Sr-deficiency in Sr_0.9_Ca_0.1_Si_2_ (Lue et al., 2013c). It thus demonstrates that the thermoelectric performance can be successfully enhanced with the introduction of appropriate amount of lattice imperfections in the Sr_0.9_Ca_0.1_Si_2_ system. Supposedly, this simple scenario can also be applied to the Sr_0.92_Y_0.08_Si_2_ alloy, and other thermoelectric systems to further enhance their *ZT* value.

## Thermoelectric performance of the optimized SrSi_2_-based alloys

### Fitted lattice thermal conductivity data of the optimized SrSi_2_-based alloys using debye equation

**Figure 15:**
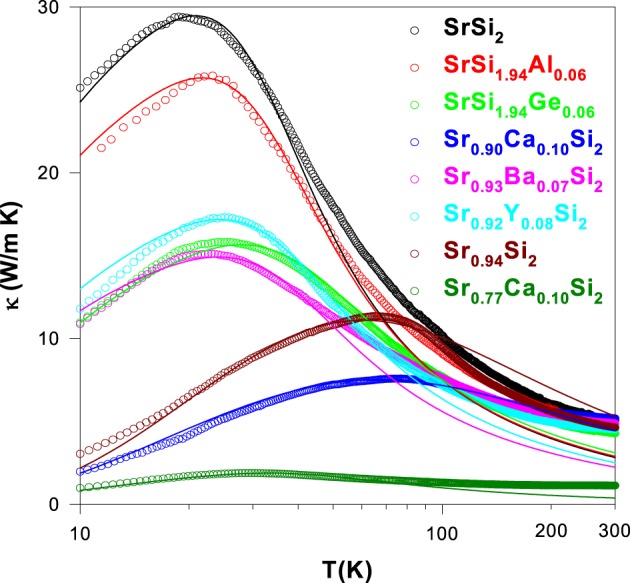
**Temperature-dependent lattice thermal conductivity of the substituted and Sr-deficient SrSi_2_ alloys**. Each solid curve represents the calculated phonon thermal conductivity.

In order to reveal the phonon-point-defect scattering effect on thermal conductivity of the SrSi_2_-based alloys, lattice thermal conductivity of the pure, substituted and Sr-deficient/Sr-rich SrSi_2_ alloys (SrSi_1.94_Al_0.06_, SrSi_1.94_Ge_0.06_, Sr_0.90_Ca_0.10_Si_2_, Sr_0.93_Ba_0.07_Si_2_, Sr_0.92_Y_0.08_Si_2_, Sr_0.94_Si_2_, and Sr_0.77_Ca_0.1_Si_2_) are evaluated by subtracting the electronic thermal conductivity (κ_*e*_) from their measured thermal conductivity (κ) using Wiedemann–Franz law as described in Section Introduction. As seen in the Figure 15, it is found that the Sr-deficient Sr_0.9_Ca_0.1_Si_2_ alloy (Sr_0.77_Ca_0.1_Si_2_) has the lowest room-temperature κ_*L*_ value of about ~1.1 W/m-K among these studied alloys presented here (Table 2). For the Sr_0.92_Y_0.08_Si_2_ alloy, the room-temperature lattice thermal conductivity is about 4.5 W/m-K, slightly smaller than that of pure SrSi_2_ (Table 2). Moreover, the low-temperature phonon peak is found to reduce drastically for Sr_0.77_Ca_0.1_Si_2_, while the Sr_0.92_Y_0.08_Si_2_ alloy shows a modest reduction in low-*T* κ_*L*_ (Figure 15) (Lue et al., 2009, 2013c). It is important note here that the room-temperature thermoelectric power factor (*S*^2^/ρ) is very high (6.4 × 10^−3^ W/m-K^2^) for Sr_0.92_Y_0.08_Si_2_ alloy (Table 2), whereas the Sr_0.77_Ca_0.1_Si_2_ alloy has a moderate *PF* value of 1.5 × 10^−3^ W/m-K^2^. Besides, the temperature-dependent lattice thermal conductivity, κ_*L*_(*T*) of these alloys is calculated using the Debye equation (described in the Section Lattice Thermal Conductivity) to analyze the influence of different substitution elements on the phonon scattering processes in the SrSi_2_-based alloys. Such an analysis was successfully employed for other silicides (Lue et al., 2004, 2013a; Kuo et al., 2005) and various other compounds in our previous reports (Ramachandran et al., 2014a,b). It is noticed that the lattice thermal conductivity of these alloys can be fitted well in the low-temperature region below 100 K, shown as solid lines in Figure 15. However, the difference between the measured and calculated data at high temperatures could arise from a number of factors, such as radiation losses during the experiments, the temperature dependence of the Lorentz number, and the undetermined Debye temperatures for the substituted alloys.

The deduced parameters of *v/L*, *A*, and *B* (using the Equations 1 and 2) of these optimized SrSi_2_-based alloys are listed in Table 3. The values of *v/L* is found to be enhanced with different substitution elements in both Sr-site and Si-site of SrSi_2_ alloy, likely due to the increase in the phonon-grain boundary scattering, as the grain size (*L*) decreases with the substitution which induces the chemical pressure in the SrSi_2_ lattice. Here, we assume that the phonon velocity is an intrinsic physical property which does not vary significantly by substitution. Besides, the free-fitting parameter *A* is also found to increase significantly for the substitution of different elements, indicating the significance of phonon-point-defect scattering in the lattice thermal transport of these substituted SrSi_2_ alloys. Generally, the grain boundary scattering dominants the low-*T* phonon thermal transport, whereas the point-defect scattering is most likely responsible for the position and shape of the phonon peak which occurs below 50 K. Therefore, we can conclude here that the change in low-*T* κ_*L*_ of these substituted and Sr-deficient alloys is essentially attributed to the modification of the phonon-point-defect scattering, according to the model proposed by Klemens (1955). This model states that the pre-factor *A* is proportional to the relative concentration of point defects. In fact, the enhanced phonon scatterings by point defects is basically originated from the mass fluctuations between host (Sr and Si) and dopant (Ba, Ca, Y, Ge, and Al) in the SrSi_2_ alloy, since the difference in their atomic radius and/or atomic mass are quite large (Table 1). In addition, other lattice defects such as vacancies can also be introduced by Sr-deficiency, which could lead to a substantial number of point defects in the Sr_1 − δ_ Ca_0.1_Si_2_, which subsequently suppress its phonon thermal conductivity.

**Table 3:** **The estimated parameters from lattice thermal conductivity (κ_*L*_) fitting of the SrSi_2_-based alloys using the Equations (1, 2)**.

**Sample**	***v*/*L* (10^6^ s^−1^)**	***A* (10^−42^ s^3^)**	***B* (10^−18^ s K^−1^)**
SrSi_2_	7.1	5.5	5.0
SrSi_1.94_Al_0.06_	7.5	6.5	4.8
SrSi_1.94_Ge_0.06_	38.0	7.0	4.0
Sr_0.90_Ca_0.10_Si_2_	1500	6.0	2.0
Sr_0.92_Ba_0.07_Si_2_	18.0	10.0	5.0
Sr_0.92_Y_0.08_Si_2_	22.0	7.5	5.5
Sr_0.94_Si_2_	2000	2.0	2.8
Sr_0.77_Ca_0.1_Si_2_	2300	23.0	52.0

### Figure-of-merit of the optimized SrSi_2_-based alloys

**Figure 16:**
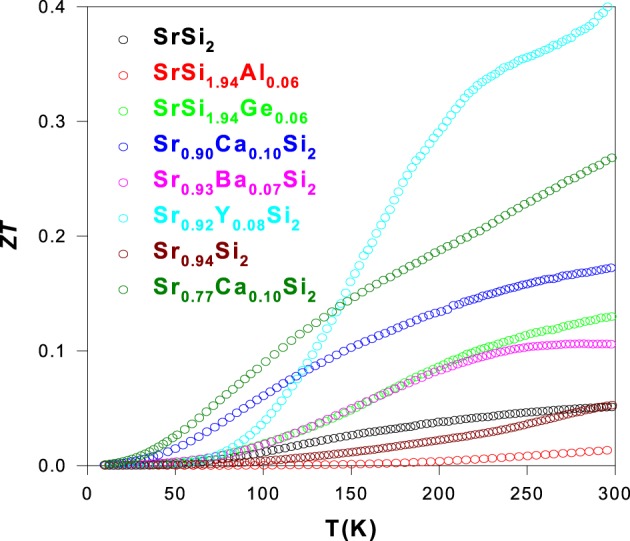
**The deduced *ZT* value vs. temperature for the substituted and Sr-deficient SrSi_2_ alloys**.

The estimated figure-of-merit, *ZT* as a function of temperature for all optimized SrSi_2_ alloys using the measured values of ρ, *S*, and κ is shown in Figure 16. It is found that a reduction in *ZT* value for the Al-substituted SrSi_2_ is observed, as a result of a considerable reduction in *S* value through hole doping (Kuo et al., 2012). On the other hand, the substitution of Ca, Ba, Y, and Ge elements in the SrSi_2_ leads to an enhancement in their *ZT* value over entire temperature range of 10–300 K (Lue et al., 2009, 2013a,b). A maximum room-temperature *ZT* value of about 0.41 has been attained for the Sr_0.92_Y_0.08_Si_2_ alloy with a large thermoelectric power factor of 6.4 × 10^−3^ W/m-K^2^, owing to the induced changes in the electronic band structure and carrier mobility through the Y doping (Lue et al., 2009). Such a *ZT* value in Sr_0.92_Y_0.08_Si_2_ is almost an order of magnitude higher than that of stoichiometric SrSi_2_ (Table 2). Besides, the second highest *ZT* value of about 0.27 at room temperature is achieved for the Sr_0.77_Ca_0.1_Si_2_ alloy (Table 2), mostly due to the combination of a substantial reduction in κ_*L*_ induced by Sr-deficiency (Lue et al., 2013c), and an enhancement in *S* value by Ca substitution (Lue et al., 2013b). It is noted that the temperature variation of *ZT* for Sr_0.77_Ca_0.1_Si_2_ increases quite linearly with temperature above 100 K. A reasonable extrapolation of the ρ, *S*, and κ data of Sr_0.77_Ca_0.1_Si_2_ to high temperatures yields a possible *ZT* value of about 0.52 at 850 K, attributed to a substantial drop in κ by the introduction of Sr-deficiency in Sr_0.9_Ca_0.1_Si_2_ (Lue et al., 2013c). In addition, a noticeable enhancement in the *ZT* value of SrSi_2_ is also obtained through Ca and Ba substitution onto the Sr sites, and Ge substitution onto the Si sites with the room-temperature *ZT* values of about 0.13, 0.17, and 0.11 for SrSi_1.94_Ge_0.06_, Sr_0.9_Ca_0.1_Si_2_, and Sr_0.93_Ba_0.07_Si_2_, respectively, (Lue et al., 2013a,b). Finally, a further reduction in κ_*L*_ of Sr_0.92_Y_0.08_Si_2_ has to be realized by the introduction of suitable Sr-deficiency level to further enhance its *ZT* value, as similar to the study on the Sr_0.9 − δ_Ca_0.1_Si_2_ systems (Lue et al., 2013c), for the practical applications.

### Applications of SrSi_2_-based silicides

In general, silicides have been widely investigated as materials for interconnections, gates in the metal-oxide-semiconductor structures, ohmic contacts, and Schottky barriers in Si-integrated circuits (Murarka, 1983; Maex and van Rossum, 1995; Miglio and d'Heurle, 2000). Recently studied semiconducting silicides have attracted attention as materials that provide new prospects for Si-based devices, such as a light-emitting diode (Leong et al., 1997; Borisenko, 2000). Moreover, the physical properties of SrSi_2_-based silicides are of great importance due to their applications in electronic devices, especially in integrated electronic circuits. In this article, we have shown that the SrSi_2_ system could be also a potential candidate for advanced thermoelectric applications through suitable chemical substitution of third elements. For example, the high thermoelectric figure-of-merit, *ZT* ~ 0.41 at room temperature has been obtained for the Sr_0.92_Y_0.08_Si_2_ alloy (Lue et al., 2009), which is nearly one order of magnitude higher than that of stoichiometric SrSi_2_. In addition, the second highest room-temperature *ZT* value (~0.27) was achieved for the Sr_0.77_Ca_0.1_Si_2_ alloy, due to the combination of a substantial reduction in κ_*L*_ induced by Sr-deficiency, and an enhancement in *S* value by Ca substitution (Lue et al., 2013c). These encouraging findings suggest that a tuning of electronic band structure by suitable alloying/substitution along with right Sr-deficiency level could leads to a significant enhancement in their thermoelectric performance (Imai et al., 2011), which could make them very useful materials in the advanced thermoelectric devices.

## Summary

Substitution/alloying of the third element in both Sr and Si sites of SrSi_2_ alloy are reviewed here to realize the optimized thermoelectric performance in the eco-friendly SrSi_2_ material. To achieve the goal, the combination of three aspects has been employed in the SrSi_2_-based alloys: (a) the reduction of the electrical resistivity by the introducing charge carriers (SrSi_2 − *x*_Al_*x*_ and Sr_1 − *x*_Y_*x*_Si_2_), (b) the enhancement of the Seebeck coefficient through the band engineering (SrSi_2 − *x*_Al_*x*_, Sr_1 − *x*_Ba_*x*_Si_2_, Sr_1 − *x*_Ca_*x*_Si_2_, and Sr_1 − *x*_Y_*x*_Si_2_), and (c) the reduction of the thermal conductivity by the introduction of point defects or lattice imperfections (all above mentioned SrSi_2_-based alloys as well as Sr_*y*_Si_2_ and Sr_0.9 − δ_Ca_0.1_Si_2_). From the studies of thermoelectric properties on these SrSi_2_-based alloys, it is found that a maximum room-temperature figure-of-merit (*ZT*) of about 0.41 is obtained for the Sr_0.92_Y_0.08_Si_2_ alloy with a highest thermoelectric power factor (*S*^2^/ρ) of 6.4 × 10^−3^ W/m-K^2^ and the lowest electrical resistivity (~0.28 mΩ cm) among all studied SrSi_2_-based alloys. In addition, the largest Seebeck coefficient (~225 μV/K) and the lowest lattice thermal conductivity (~1.1 W/m-K) at room temperature are obtained for the Sr_0.90_Ca_0.10_Si_2_ (*ZT* = 0.17) and Sr_0.77_Ca_0.1_Si_2_ (*ZT* = 0.27) samples, respectively. We thus demonstrated here that the thermoelectric performance of the eco-friendly SrSi_2_ alloy can be effectively enhanced by several approaches, i.e., lowering the electrical resistivity by doping charge carriers, enhancing the Seebeck coefficient by the band engineering via chemical pressure, and lowering the lattice thermal conductivity by introducing point defects or lattice imperfections. In conclusion, the substantial reduction in κ_*L*_ of the SrSi_2_ alloy above 200 K has to be realized by the introduction of Sr-deficiency or co-doping of Ca with Y into Sr sites of SrSi_2_ to further improve its *ZT* value to make it a suitable material for possible future thermoelectric applications.

### Conflict of interest statement

The authors declare that the research was conducted in the absence of any commercial or financial relationships that could be construed as a potential conflict of interest.
